# Supplementary material for: Patterns of Intron Gain and Loss in Fungi
Source: PLoS Biol. 2004 Nov 30;2(12):e422. doi: 10.1371/journal.pbio.0020422 (PMC532390; doi:10.1371/journal.pbio.0020422)
Supplement: Table S1 — Also available at http://genes.mit.edu/NielsenEtAl/. (4.3 MB ZIP). [file pbio.0020422.st001.zip › NielsenEtAl/html/1350.html]

AN0359.1.NCU02208.1.MG01978.1.FG05777.1


```
 CLUSTAL W (1.82) Multiple Sequence Alignments - Introns Inserted


Sequence 1: NCU02208.1	747 aa
Sequence 2: MG01978.1	746 aa
Sequence 3: FG05777.1	746 aa
Sequence 4: AN0359.1	738 aa
Alignment Length: 750 aa
Number Identitical Residues: 403 aa
Alignment Score (without introns) 20814


MG01978.1 	MAPSYEHLREADLDEDEFDEDEVDVSD~LREKFEVQLEQGFDTFVVIDGLPEVTEEQKPK
NCU02208.1	MAPSFDHLRDEDLDEDDFDVDEVDISD~IREKYEVQLEQGYDAFVVVDGLPEVNEEQKPK
FG05777.1 	MAPSFDHLREADLDDDEFNDDDIDISD~LRERFEVSLDQGYDNFVVIDGLPQVTEDQKPK
AN0359.1  	MAPSFENLSEQDLHEE--EEEEIDFSD1LKAQYEVKLEEGLDTFVVIDGLPVVPEENRQK
          	****:::* : **.::  : :::*.** :: ::**.*::* * ***:**** * *::: *

MG01978.1 	LVKFLLKKLTSVGKTKEDMIDMPMGPDGKSLR2FAFVEYSSPGEAAAAVRQLDRVPLDKK
NCU02208.1	LVKFLLKKLNTVGKTREDLIFMPMGEDGKSLR2FAFVEYSSPAEAAAACRQLDLVPLDKK
FG05777.1 	LVKFLLKKLNTVGKTREDSVHMPMGDDGKSLR2FAFVEYSSPAEAAAATRQLDLTPLDKK
AN0359.1  	LIKFLLRKLNTVGHTSEDAVFMPLNEKNMSEG2FAFVEYETAEQAVAAVKQLHGTPLDKK
          	*:****:**.:**:* ** : **:. .. *   ******.:. :*.** :**. .*****

MG01978.1 	HTLRVNKLMDVDRFGREGRIDDEYQPPHIDEFHPRDHLRSFMADPSGRGRDQFVMFRGDH
NCU02208.1	HTLRVNKLTDVDRYGREGRIDDEYTPPKIEEFQEKEHLRSFMADPSGRGRDQFVMFRGES
FG05777.1 	HTLRVNKFTDIERYGREGRIDETYQPPQIEDFTEKEHLRWFLKDPSGRGRDQFVMYRGDS
AN0359.1  	HTLLVNKLMDIERYGREGRIDEEYKPPNIEPFTEKEHLRSWLGDPN--ARDQFALYRGDK
          	*** ***: *::*:*******: * **:*: *  ::*** :: **.  .****.::**: 

MG01978.1 	VGVFWNNEKDTPENIVDRPNW~TESFVQWSPLGTYLLSMHMQGVQLWGGPKWDRLGRFPH
NCU02208.1	VGVFWNNEKDTPENIVDRQHW~TETFVQWSPLGTYLTSVHAQGVQLWGGPSWTRQRRFAH
FG05777.1 	VGIYWNNEKDQPENIVERPFW2TEAFIQWSPLGTYLTSVHAQGVQLWGGASWSRQARFAH
AN0359.1  	VGVFWNNKSNPPENVVDRAHW~TQLFVQWSPKGTYLASVHPQGVQLWGGPTFSKQKQFPH
          	**::***:.: ***:*:*  * *: *:**** **** *:* ********..: :  :*.*

MG01978.1 	PFVNMAAFSPQENYLVTWSNRPISIPDEGHPALSMDDDGKNYVIWDIATGKPLRSFANLD
NCU02208.1	PFVNLVAFSPNEKYLVTWSNRPISIPEEGHPALSVEDDGKNYVIWDIETSKPLRSFAQLD
FG05777.1 	PFVNMISFSPGEKYIVTWSNRPISIPDEGHPALSIDDHGKNYVIWDIATATPLRSFANLE
AN0359.1  	PFVSLIEFSPGESYLTTWSARPIQV-EEGHPVLTYEEDGKNIIVWDIVTGKPLRSFVSHD
          	***.:  *** *.*:.*** ***.: :****.*: ::.*** ::*** *..*****.. :

MG01978.1 	LPPDDPNKPPR---KHPWPAFKWSSDDKYVARLTQGQSISVYELPRMNLLDKTTIKVEGV
NCU02208.1	TPAAAEGEAPKKAPKFPWPAFKWSADDKYVARLNPGQSISVYELPRMNLLDKTAIKIEGV
FG05777.1 	PPKVEDGKPPP---KMQWPAFKWSADDKYVARLNPGQSISVYELPRMNLLDKTSIKIDGV
AN0359.1  	LAGPESEAQPKK--KVQWPAFKWSADEKYVARMLQGQSISIYELPRMNLLGKTSVKIDGV
          	 .       * .  *  *******:*:*****:  *****:*********.**::*::**

MG01978.1 	QDFEWAPSRPQRDGVKTYEQMFCYWTPEIGSNPAKVGLMSIPSKEVVRSLNLFSVSDVKL
NCU02208.1	VDFDWAPATVQRDGVKSYEQLFCFWTPEIGSNPARVGLMSIPSKQVVRSLNLFSVSDAKL
FG05777.1 	VDFDWAPATPKRDGVKEYEQLFCYWTPEIGSNPAKVGLMSVPSKEVVRSLNLFSVSDAKL
AN0359.1  	MDFEWSPATVNRDGVKQYEQLLCFWTPEIGSNPARVALMSVPSKEIVRTRNLFNVSDVKL
          	 **:*:*:  :***** ***::*:**********:*.***:***::**: ***.***.**

MG01978.1 	HWQSEGAYLCVKVDRHSKSKKSQATTLEIFRVKEKGVPVEVVDTIKDTVINFAWEPKGDR
NCU02208.1	HWQSEGAYLCVKVDRHSKSKKSQATTLEIFRVKEKGVPVEVVDTIKDTVINFAWEPKGDR
FG05777.1 	HWQSDAAYICVKVDRHSKSKKSQATTLEIFRVKEKGVPVEVVDTIKDTVINFAWEPKGDR
AN0359.1  	HWQSQGNFVCVKVDRHSKSKKSMATNLEIFRVREKGVPVEVVDSLKDTVINFSWEPNGAR
          	****:. ::************* **.******:**********::*******:***:* *

MG01978.1 	FLIITTVTPTGEVAVQPKTAISFFCPEKSK-GSTVGNFKHLRTLDKRNSNAIYWSPKGRF
NCU02208.1	FVIITTTEPVGATAVPPKTSVAFFCPEKAK-GNAVGNFKHLRTLEKKNSNAIYWSPKGRF
FG05777.1 	FAIISTTEPVGVTAVAPKTAVSFFCPEKAKKGQQVGNFKHLRTLDKKNSNAIYWSPRGRF
AN0359.1  	FVLITTGDASG-AGAPPKTAVSFFAPEKKG--VQAGNFKLVRTIEKKTSNAIYWSPKGRF
          	* :*:*  . * ... ***:::**.***      .**** :**::*:.********:***

MG01978.1 	VVVATVHNQNSSDLDFFDLDFEGEKPEGEKDLTANLQLMNTADHYGITDVEWDPSGRFVA
NCU02208.1	VVVATVANTQSSDLDFYDLDFEGEKPESDKDLTANLQLMNTADHYGVTDVEWDPSGRFVA
FG05777.1 	VVIATIANTQSSDLDFFDLDFEGEKPESDKDLTANLQAMNTADHYGVTDVEWDPSGRFVA
AN0359.1  	VVVATVHSQSNFDLDFWDMDFEGEKNDNEKDLAANLQLMKTVDHYGVTDIEWDPTGRYVV
          	**:**: . .. ****:*:****** :.:***:**** *:*.****:**:****:**:*.

MG01978.1 	TWASAWKHT0MENGYHMYDFKGEQLREEAVEKFKQLQWRPRPPTLLAKEEQKQIRKNLRE
NCU02208.1	TWASAWKHS0MENGYHLYDFKGEQLREEAIEKFKQFQWRPRPATLLSKEEQKAIRRNLRE
FG05777.1 	SWASAWKHS0MENGYHIYDFKGEALREEPLEKFKQFAWRPRPATLLTKEEQKAVRKNLRE
AN0359.1  	SGASAWTHQ0MENGFNLHTFSGETLAENPTDKFKQFLWRPRPPTLLSKEEQKQVRKNLRE
          	: ****.*  ****:::: *.** * *:. :****: *****.***:***** :*:****

MG01978.1 	YSRVFEQEDAERIAGADQEVVDNRRRILEDWYEWRESVDVEVDEESAAMGVSSNPAEELL
NCU02208.1	YSRIFEQEDAERISSADVAVVEARRRLLEEWFAWREAIRQEVAEEREIYGLPADPVADLI
FG05777.1 	YSRVFEQEDADRGASADLAVVEARRRMLEEWHSWRESMEADVLEEREVLGLPKDPHAPLL
AN0359.1  	YSKEFEEEDKYAVDIANTAVVEKRKRVLNEWVAWLKREKELLTEEKEAYGLP-EEADQPK
          	**: **:**      *:  **: *:*:*::*  * :     : **    *:. :      

MG01978.1 	KAKTAEILASGQEEEQVIEEIVEEVLEESEEIVS
NCU02208.1	KAKTPELATD--QEEQVIEEIMEEVLEETEEIVQ
FG05777.1 	EAYTKSLDTAS-DEDRVIEEIVEEVIEESEEVLA
AN0359.1  	AAKDAPTNTED-KGETVVEEIVEEIVEESEEIIG
          	 *      : . . : *:***:**::**:**::
```
